# Emergency groin hernia repair: implications in elderly

**DOI:** 10.1186/1471-2482-13-S2-S29

**Published:** 2013-10-08

**Authors:** Rita Compagna, Roberto Rossi, Francesca Fappiano, Tommaso Bianco, Antonello Accurso, Michele Danzi, Salvatore Massa, Giovanni Aprea, Bruno Amato

**Affiliations:** 1 Department of Clinical Medicine and Surgery, University of Naples Federico II, Via S. Pansini, 5 - 80131 Napoli, Italy

## Abstract

**Background:**

Groin hernia is one of the most frequently encountered pathologies occurring in old age and it is often the cause of emergency procedures. In our study we evaluate the impact of emergency procedures in over 75 patients compared to younger patients.

**Methods:**

We conducted a retrospective study about patients who underwent emergency hernioplasty between September 2007 and January 2013. Bilateral hernias and recurrences were excluded. We divided patients into two groups by age (under and over 75 years old) and then analyzed the early postoperative surgical complications.

**Results:**

A total of 48 patients were enrolled, 18 were included in under 75 group and 30 in over 75. In the older group we found a higher rate of comorbidity and also a significant higher rate of postoperative complications. Two patients of over 75 group died.

**Conclusions:**

Our data suggests that a quick diagnosis and elective surgical procedures are desirable in order to avoid the complications that occur in emergency operations.

## Background

Groin hernia is more frequent in elderly patients, conditions which increase intraabdominal pressure and the loss of strength of the abdominal wall, play a determinant rule in that [1-4]. In fact the abdominal wall increasing is due to the presence of constipation, prostatism, bronchitis (coughting) or abdominal fat deposit that may affect old patients, furthermore the loss of strength of the abdominal wall is caused by alteration in collagen (the collagen becomes more rigid and crystalline and its tension diminished) typical of elderly [2,5,6]. Also the ageing population is increasing, with a corresponding increasing demand for surgical services [7]. Elective groin hernia repair is a surgical procedure with good results and minimal morbidity and recent studies suggest that it is quite safe [8-11,16,17]. Despite this is still widely thought that in elderly is better a conservative approach [7,12], but excessive waiting times for elective repair increase the risk of strangulation, bowel resection and mortality especially in older patients[13,14]. Also the higher rate of comorbidity in combination with the usage of general anesthesia in old patient increases the risk of complications [7,15]. In our study we evaluate the impact of emergency groin hernia repair in over 75 patients compared to younger patients.

## Methods

In this retrospective study we included patients who underwent emergency hernioplasty in our department in the period between September 2007 and January 2013. We divided patients into two age classes (under and over 75 years old). Exclusion criteria were: previous surgery for groin hernia, bilateral inguinal hernia, connective tissue disease, immunocompromised host. For incarceration we intend irreducibility of a groin hernia and for strangulation we intend a irreducible hernia with objective signs of ischemia or gangrene. General anesthesia was induced and maintained in all patients using standard techniques. Perioperative intravenous antibiotic prophylaxis was given, usually cefazoline, and antibiotics were then continued orally until the end of the first postoperative week in all patients[4,14]. The Lichetenstein hernioplasty was done by resident surgeons[18,19]. Patients were discharged when they were ambulating with minimal pain and had no fever. Follow-up was made at 1 week and 4 weeks postoperatively.

## Results

A total of 48 patient were enrolled, 18 were included in under 75 group and 30 in over 75. Thus, 14 patients were male and 9 were female. In 17 cases we treated an incarceration, in 25 cases a strangulation and 6 cases required a bowel resection (5 of them in the elderly group). Comorbidity found were higher in over 75 group (Table 1) and the mean were hypertension (15 cases in under 75 and 27 cases in over 75), coronary disease (11 cases in under 75 and 23 in over 75) and benign prostatic hypertrophy (5 cases in under 75 and 18 in over 75). In the older group we found also a significant higher rate of postoperative complications (Table 2). The most frequent complications were mesh infection (5 cases in under 75 and 18 in over 75), urinary infection (9 cases in under 75 and 20 in over 75) and wound infection (8 cases in under 75 and 12 in over 75). We also had a relevant number of wound hematoma, severe bleeding and respiratory disease. Furthermore two patients of over 75 group died.

**Table 1:** 

Patients n. 48			
**Age**		**Under 75** **18 (37.5%)**	**Over 75** **30 (62.5%)**

Sex	Male Female	14 (29.1%) 9 (18.75%)	18 (37.5%) 7(14.6%)

Comorbidity	Hypertension C D COPT Diabetes BPH Stroke KD	15(83.3%) 11(61.1%) 3(16.7%) 10(55.5%) 5(27.7%) 0 1(5.5%)	27 (90%) 23 (76.6%) 9(30%) 8(26.7%) 18(60%) 1(3.3%) 2(6.6%)

Emergency Treated	Incarceration Strangulation Bowel-resection	10 (55.5%) 7(38.9%) 1(5.5%)	7 (23.3%) 18(60%) 5(16.6%)

**Table 2:** Complications

Patients n. 48		
**Age**	**Under 75** **18 (37.5%)**	**Over 75** **30 (62.5%)**

Wound infection	8 (44.4%)	12 (40%)
Wound hematoma	5 (27.7%)	9 (30%)
Testicular swelling	3 (16.6%)	2 (6.6%)
Mesh infection	5(27.7%)	18(60%)
Severe bleeding Urinary infections Peritonitis Respiratory disease Ischemic heart disease Neurological disease	4(22.2%) 9(50%) 0 5(27.7%) 2(11.1%) 0	14(46.6%) 20(66.6%) 1(3.3%) 10(33.3%) 3(10%) 1(3.3%)
Death	0	2 (6.6%)

## Conclusions

In conclusion, our data suggest that acutely incarcerated or strangulated groin hernia in elderly is a serious problem because of the high morbidity and mortality rate found in the over 75 group that are, in our opinion, unacceptable. So quick diagnosis and elective surgical procedures are desirable in order to avoid the complications that occur in emergency operations, moreover elderly patients should be warned about the potential danger of groin hernia emergency repair.

## List of abbreviations

ASA: American Society of Anaesthesiologists; COPD: chronic obstructive pulmonary disease; BPH: benign prostatic hypertrophy; CD: cardiovascular disease;

KD: kidney disease.

## Competing interests

The authors declare that they have no competing interests.

## Authors' contributions

RC: conception and design, interpretation of data, given final approval of the version to be published. RR, FF, TB, AA, MD, GA: acquisition of data, drafting the manuscript, given final approval of the version to be published. SM, BA: conception and design, given final approval of the version to be published.

## Authors' information

RC: Post-Graduate Doctorate in Vascular Surgery at University "Federico II" of Naples. RR, FF, TB: Resident in General Surgery Training Programme at University "Federico II" of Naples. AA, MD, GA: Aggregate Professor of Surgery at University "Federico II" of Naples, Italy. SM: Full Professor of Surgery at University "Federico II" of Naples, Italy.

BA: Associate Professor of Surgery at University "Federico II" of Naples, Italy.

## Declarations

Funding for this article came from university funds.

This article has been published as part of *BMC Surgery *Volume 13 Supplement 2, 2013: Proceedings from the 26th National Congress of the Italian Society of Geriatric Surgery. The full contents of the supplement are available online at http://www.biomedcentral.com/bmcsurg/supplements/13/S2
